# Diaqua­bis­(1,10-phenanthroline)nickel(II) tetra­kis­(cyanido-κ*C*)nickelate(II) tetra­hydro­furan solvate monohydrate

**DOI:** 10.1107/S1600536810036974

**Published:** 2010-09-18

**Authors:** Zhi-li Fang, Jun Wang

**Affiliations:** a School of Basic Science, East China Jiaotong University, Nanchang 330013, People’s Republic of China; b Zhongshan Polytechnic, Zhongshan, Guangdong 528404, People’s Republic of China

## Abstract

The title complex, [Ni(C_12_H_8_N_2_)_2_(H_2_O)_2_][Ni(CN)_4_]·C_4_H_8_O·H_2_O, consists of a cationic [Ni(C_12_H_8_N_2_)_2_(H_2_O)_2_]^2+^ unit, an anionic [Ni(CN)_4_]^2−^ unit, one uncoordinated water and one tetra­hydro­furan mol­ecule. In the cationic unit, the Ni^2+^ atom is coordinated by four N atoms and two O atoms from two 1,10-phenanthroline ligands and two water mol­ecules in a distorted octa­hedral coordination environment. In the anionic unit, the Ni^2+^ atom is in a square-planar coordination by four C atoms from four monodentate terminal cyanide ligands. O—H⋯N and O—H⋯O hydrogen bonds link neighboring cationic and anionic units, forming a three-dimensional supra­molecular network. The inter­stitial tetra­hydro­furan mol­ecule is independently disordered over two sites in a 1:1 ratio.

## Related literature

For general background to cyanido–metal complexes, see: Miyasaka *et al.* (2007 ▶); Shatruk *et al.* (2009 ▶); Kou *et al.* (2001 ▶); Paharova *et al.* (2003 ▶); Yuge *et al.* (1996 ▶); Yun *et al.* (2004 ▶).
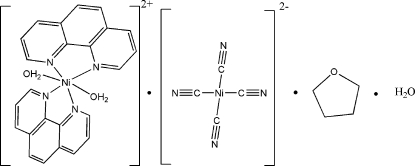


## Experimental

### 

#### Crystal data

[Ni(C_12_H_8_N_2_)_2_(H_2_O)_2_][Ni(CN)_4_]·C_4_H_8_O·H_2_O
*M* *_r_* = 708.06
Monoclinic, 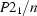

*a* = 11.4623 (3) Å
*b* = 14.3184 (4) Å
*c* = 19.2329 (4) Å
β = 91.189 (2)°
*V* = 3155.86 (14) Å^3^
*Z* = 4
Mo *K*α radiation
μ = 1.24 mm^−1^
*T* = 296 K
0.25 × 0.23 × 0.18 mm

#### Data collection

Bruker APEXII CCD area-detector diffractometer
Absorption correction: multi-scan (*SADABS*; Sheldrick, 1996 ▶) *T*_min_ = 0.746, *T*_max_ = 0.807
29619 measured reflections
6199 independent reflections
3756 reflections with *I* > 2σ(*I*)
*R*_int_ = 0.098

#### Refinement

*R*[*F* ^2^ > 2σ(*F* ^2^)] = 0.048
*wR*(*F* ^2^) = 0.119
*S* = 1.02
6199 reflections
436 parameters
70 restraints
H-atom parameters constrained
Δρ_max_ = 0.36 e Å^−3^
Δρ_min_ = −0.39 e Å^−3^

### 

Data collection: *APEX2* (Bruker, 2004 ▶); cell refinement: *SAINT* (Bruker, 2004 ▶); data reduction: *SAINT*; program(s) used to solve structure: *SHELXS97* (Sheldrick, 2008 ▶); program(s) used to refine structure: *SHELXL97* (Sheldrick, 2008 ▶); molecular graphics: *XP* in *SHELXTL* (Sheldrick, 2008 ▶); software used to prepare material for publication: *SHELXTL*.

## Supplementary Material

Crystal structure: contains datablocks I, global. DOI: 10.1107/S1600536810036974/zl2307sup1.cif

Structure factors: contains datablocks I. DOI: 10.1107/S1600536810036974/zl2307Isup2.hkl

Additional supplementary materials:  crystallographic information; 3D view; checkCIF report

## Figures and Tables

**Table 1:** Hydrogen-bond geometry (Å, °)

*D*—H⋯*A*	*D*—H	H⋯*A*	*D*⋯*A*	*D*—H⋯*A*
O4—H4*A*⋯N8^i^	0.84	2.02	2.857 (6)	173
O2—H2*A*⋯O4^ii^	0.82	2.30	3.116 (5)	173
O2—H2*B*⋯N5^iii^	0.79	2.60	3.154 (5)	128
O1—H1*B*⋯N5^iii^	0.86	2.63	3.377 (6)	147
O1—H1*B*⋯N5^iii^	0.86	2.63	3.377 (6)	147
O1—H1*A*⋯N6^iv^	0.90	2.39	3.250 (5)	161
O4—H4*B*⋯N7	0.83	1.99	2.804 (5)	167

Symmetry codes: (i) 
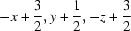
; (ii) 
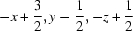
; (iii) 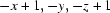; (iv) 
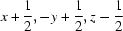
.

**Fig. 1.**
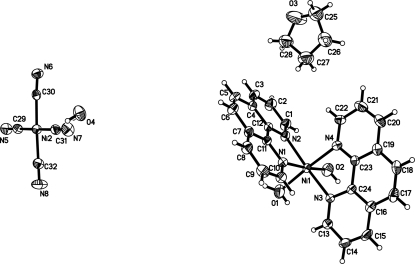
The structure of the title compound, showing the atomic numbering scheme. Non-H atoms are shown with 30% probability displacement ellipsoids. The disordered section is omitted for clarity.

**Fig. 2.**
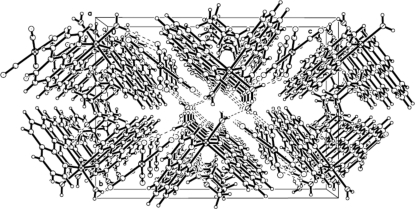
View of the three-dimensional structure of the title compound, view down the direction of the a-axis. Dashed lines indicate hydrogen bonds.

## supplementary crystallographic information

### Comment

The study of cyanide-bridged complexes has gained great recognition over the last decade not only owing to their fascinating structural diversity and their intriguing topological networks, but also because of interesting magnetic properties, such as spin-crossover behaviour or the formation of single-molecular or single-chain magnets (Miyasaka *et al.*, 2007; Shatruk *et al.*, 2009). To date, much effort has been invested to construct cyanide-based complexes by the choice of versatile metal cyanide or cyanide-based building units (Kou *et al.*, 2001; Yun *et al.*, 2004; Yuge *et al.*, 1996). In this context we have chosen nickel cyanide as a potential bridging building block, and 1,10-phenanthroline as an auxilary ligand to construct new structures. Reaction of the two building blocks yielded the title compound [Ni(C_12_H_8_N_2_)_2_(H_2_O)_2_].[Ni(CN)_4_].C_4_H_8_O.H_2_O.

As depicted in Fig. 1, the structure of the title compound, consists of a cationic [Ni(C_12_H_8_N_2_)_2_(H_2_O)_2_]^2+^ unit, an anionic [Ni(CN)_4_]^2-^ unit, and each one interstitial water and tetrahydrofuran molecule. Thus no cyano bridged complex with different nickel centers was formed but the nickel atoms are found in separate anionic and a cationic complex ions. In the cationic unit, the six-coordinate octahedral Ni^2+^ center is surrounded by four N atoms and two O atoms from two 1,10-phenanthroline ligands and two water molecules. In the anionic unit, the square planar Ni^2+^ center is coordinated by four C atoms from four mono-dentate terminal cyanide ligands. Similar structures containing Ni(CN)_4_ units have been observed in other complexes (Paharova *et al.*, 2003). O—H···N and O—H···O hydrogen bonds (Table 1) are formed between the cationic units, the anionic units and the uncoordinating water molecules which assemble them to form a three-dimensional supramolecular network (Fig. 2). The network is also stabilized by π-π stacking interactions between the Ni(CN)_4_ units and the 1,10-phenanthroline ligands. The interplanar distance between them is ca. 3.60 Å (symmetry operator for the 1,10-phenanthroline ligand: 0.5+*x*, 0.5-*y*, -0.5+*z*). The interstitial tetrahydrofuran molecule is independently disordered over two sites in a one to one ratio (see refinement section for details).

### Experimental

Nickel cyanide (0.1107 g, 1 mmol) and 1,10-phenanthroline (0.1801 g, 1 mmol) were added to a mixture of water (10 mL) and tetrahydrofuran (5 mL). The resultant mixture was sealed in a 25 ml stainless steel reactor with a Teflon liner and kept under autogenous pressure at 413 K for 24 h, and then cooled to room temperature at a rate of 0.5 K/min. Green block shaped crystals of the title compound suitable for single-crystal X-ray diffraction analysis formed with a yield of approximately 56% based on 1,10-phenanthroline.

### Refinement

The tetrahydrofuran molecules are arranged as symmetry related pairs around a center of inversion. In the original refinement the oxygen atoms of the tetrahydrofuran molecules showed significantly elongated thermal ellipsoids indicating disorder. The THF molecule was thus refined as being disordered over two sites in a one to one ratio. Due to the significant overlap of the disordered atoms the following restraints and constraints were applied: The adps of the disordered atoms were restrained to be close to isotropic and those of equivalent atoms were set to be identical.

All water H atoms were tentatively located in difference density Fourier maps and were refined with O–H distance restraints of 0.83 (1) Å and with U_iso_(H) = 1.5 U_eq_(O). In the last stage of the refinement, they were treated as riding on their parent O atoms. All H atoms attached to C atoms were fixed geometrically and treated as riding with C—H = 0.93 Å (aromatic) or 0.97Å (tetrahydrofuran ring) and U_iso_(H) = 1.2U_eq_(C).

### Crystal data

**Table:**

[Ni(C_12_H_8_N_2_)_2_(H_2_O)_2_][Ni(CN)_4_]·C_4_H_8_O·H_2_O	*F*(000) = 1464
*M**_r_* = 708.06	*D*_x_ = 1.490 Mg m^−^^3^
Monoclinic, *P*2_1_/*n*	Mo *K*α radiation, λ = 0.71073 Å
Hall symbol: -P 2yn	Cell parameters from 4500 reflections
*a* = 11.4623 (3) Å	θ = 1.3–28.0°
*b* = 14.3184 (4) Å	µ = 1.24 mm^−^^1^
*c* = 19.2329 (4) Å	*T* = 296 K
β = 91.189 (2)°	Block, green
*V* = 3155.86 (14) Å^3^	0.25 × 0.23 × 0.18 mm
*Z* = 4	

### Data collection

**Table:**

Bruker APEXII CCD area-detector diffractometer	6199 independent reflections
Radiation source: fine-focus sealed tube	3756 reflections with *I* > 2σ(*I*)
graphite	*R*_int_ = 0.098
φ and ω scan	θ_max_ = 26.0°, θ_min_ = 1.8°
Absorption correction: multi-scan (*SADABS*; Sheldrick, 1996)	*h* = −14→13
*T*_min_ = 0.746, *T*_max_ = 0.807	*k* = −17→15
29619 measured reflections	*l* = −23→23

### Refinement

**Table:**

Refinement on *F*^2^	Primary atom site location: structure-invariant direct methods
Least-squares matrix: full	Secondary atom site location: difference Fourier map
*R*[*F*^2^ > 2σ(*F*^2^)] = 0.048	Hydrogen site location: inferred from neighbouring sites
*wR*(*F*^2^) = 0.119	H-atom parameters constrained
*S* = 1.02	*w* = 1/[σ^2^(*F*_o_^2^) + (0.0447*P*)^2^ + 1.097*P*] where *P* = (*F*_o_^2^ + 2*F*_c_^2^)/3
6199 reflections	(Δ/σ)_max_ = 0.001
436 parameters	Δρ_max_ = 0.36 e Å^−^^3^
70 restraints	Δρ_min_ = −0.39 e Å^−^^3^

### Special details

**Table:**

Geometry. All esds (except the esd in the dihedral angle between two l.s. planes) are estimated using the full covariance matrix. The cell esds are taken into account individually in the estimation of esds in distances, angles and torsion angles; correlations between esds in cell parameters are only used when they are defined by crystal symmetry. An approximate (isotropic) treatment of cell esds is used for estimating esds involving l.s. planes.
Refinement. Refinement of F^2^ against ALL reflections. The weighted R-factor wR and goodness of fit S are based on F^2^, conventional R-factors R are based on F, with F set to zero for negative F^2^. The threshold expression of F^2^ > 2sigma(F^2^) is used only for calculating R-factors(gt) etc. and is not relevant to the choice of reflections for refinement. R-factors based on F^2^ are statistically about twice as large as those based on F, and R- factors based on ALL data will be even larger.

### Fractional atomic coordinates and isotropic or equivalent isotropic displacement parameters (Å^2^)

**Table:**

	*x*	*y*	*z*	*U*_iso_*/*U*_eq_	Occ. (<1)
Ni1	0.73838 (4)	0.17737 (3)	0.04274 (2)	0.03482 (15)	
Ni2	0.48209 (5)	0.20766 (4)	0.79200 (3)	0.04305 (17)	
C1	0.4836 (4)	0.1426 (3)	0.0908 (2)	0.0572 (12)	
H1	0.4828	0.0911	0.0613	0.069*	
C2	0.3846 (4)	0.1617 (4)	0.1285 (3)	0.0663 (14)	
H2	0.3194	0.1232	0.1242	0.080*	
C3	0.3831 (4)	0.2367 (4)	0.1717 (2)	0.0600 (13)	
H3	0.3171	0.2498	0.1973	0.072*	
C4	0.4824 (4)	0.2947 (3)	0.1775 (2)	0.0462 (11)	
C5	0.4898 (4)	0.3746 (3)	0.2216 (2)	0.0576 (13)	
H5	0.4256	0.3917	0.2475	0.069*	
C6	0.5879 (5)	0.4254 (4)	0.2262 (2)	0.0605 (13)	
H6	0.5898	0.4781	0.2546	0.073*	
C7	0.6894 (4)	0.4006 (3)	0.1886 (2)	0.0453 (11)	
C8	0.7965 (4)	0.4469 (3)	0.1947 (2)	0.0558 (12)	
H8	0.8040	0.4991	0.2232	0.067*	
C9	0.8898 (4)	0.4154 (3)	0.1589 (2)	0.0576 (12)	
H9	0.9619	0.4447	0.1641	0.069*	
C10	0.8768 (4)	0.3390 (3)	0.1145 (2)	0.0449 (10)	
H10	0.9406	0.3194	0.0892	0.054*	
C11	0.6845 (3)	0.3236 (3)	0.14461 (19)	0.0374 (9)	
C12	0.5792 (3)	0.2690 (3)	0.13842 (19)	0.0385 (10)	
C13	0.9954 (4)	0.1386 (3)	0.0009 (2)	0.0487 (11)	
H13	0.9990	0.0966	0.0378	0.058*	
C14	1.0949 (4)	0.1500 (3)	−0.0389 (2)	0.0572 (12)	
H14	1.1623	0.1163	−0.0286	0.069*	
C15	1.0920 (4)	0.2110 (4)	−0.0931 (2)	0.0600 (13)	
H15	1.1574	0.2193	−0.1202	0.072*	
C16	0.9890 (4)	0.2616 (3)	−0.1077 (2)	0.0491 (11)	
C17	0.9790 (5)	0.3292 (4)	−0.1628 (2)	0.0605 (13)	
H17	1.0429	0.3416	−0.1904	0.073*	
C18	0.8782 (5)	0.3746 (3)	−0.1747 (2)	0.0590 (13)	
H18	0.8738	0.4185	−0.2103	0.071*	
C19	0.7774 (4)	0.3571 (3)	−0.1341 (2)	0.0473 (11)	
C20	0.6690 (5)	0.4007 (3)	−0.1461 (2)	0.0621 (13)	
H20	0.6601	0.4448	−0.1813	0.074*	
C21	0.5770 (5)	0.3779 (4)	−0.1056 (3)	0.0673 (14)	
H21	0.5043	0.4052	−0.1137	0.081*	
C22	0.5928 (4)	0.3134 (3)	−0.0518 (2)	0.0520 (12)	
H22	0.5296	0.2993	−0.0242	0.062*	
C23	0.7853 (4)	0.2925 (3)	−0.07893 (19)	0.0381 (9)	
C24	0.8938 (3)	0.2446 (3)	−0.0653 (2)	0.0373 (10)	
C29	0.4221 (4)	0.1348 (3)	0.8642 (2)	0.0472 (11)	
C30	0.3446 (4)	0.2780 (3)	0.7844 (2)	0.0446 (10)	
C31	0.5426 (4)	0.2827 (3)	0.7224 (2)	0.0491 (11)	
C32	0.6215 (4)	0.1392 (4)	0.7953 (2)	0.0558 (12)	
N1	0.7769 (3)	0.2932 (2)	0.10702 (15)	0.0370 (8)	
N2	0.5797 (3)	0.1947 (2)	0.09474 (16)	0.0420 (8)	
N3	0.8970 (3)	0.1838 (2)	−0.01070 (15)	0.0364 (8)	
N4	0.6931 (3)	0.2713 (2)	−0.03846 (17)	0.0402 (8)	
N5	0.3878 (3)	0.0904 (3)	0.9091 (2)	0.0645 (11)	
N6	0.2622 (4)	0.3222 (3)	0.7757 (2)	0.0596 (10)	
N7	0.5781 (4)	0.3302 (3)	0.6793 (2)	0.0723 (13)	
N8	0.7067 (4)	0.0987 (4)	0.7970 (3)	0.0916 (16)	
O1	0.8002 (3)	0.0868 (2)	0.12306 (16)	0.0744 (10)	
H1A	0.7713	0.1075	0.1634	0.112*	
H1B	0.7781	0.0298	0.1231	0.112*	
O2	0.6793 (3)	0.0641 (2)	−0.01813 (16)	0.0699 (9)	
H2B	0.6433	0.0173	−0.0162	0.105*	
H2A	0.7353	0.0480	−0.0414	0.105*	
O4	0.6238 (3)	0.5037 (2)	0.61803 (16)	0.0754 (10)	
H4B	0.6104	0.4566	0.6421	0.113*	
H4A	0.6749	0.5274	0.6444	0.113*	
C25	0.158 (5)	0.469 (3)	−0.007 (2)	0.102 (8)	0.50
H25A	0.1659	0.5367	−0.0027	0.122*	0.50
H25B	0.0971	0.4568	−0.0411	0.122*	0.50
C26	0.272 (5)	0.429 (4)	−0.032 (3)	0.105 (6)	0.50
H26A	0.2602	0.3880	−0.0713	0.126*	0.50
H26B	0.3279	0.4773	−0.0426	0.126*	0.50
C27	0.304 (6)	0.377 (4)	0.033 (3)	0.097 (8)	0.50
H27A	0.3687	0.4085	0.0569	0.116*	0.50
H27B	0.3302	0.3146	0.0211	0.116*	0.50
C28	0.202 (4)	0.370 (4)	0.082 (3)	0.099 (7)	0.50
H28A	0.1682	0.3080	0.0806	0.119*	0.50
H28B	0.2253	0.3853	0.1290	0.119*	0.50
O3	0.125 (2)	0.4346 (14)	0.0555 (12)	0.149 (8)	0.50
C26'	0.264 (5)	0.448 (4)	−0.024 (3)	0.105 (6)	0.50
H26C	0.2788	0.4398	−0.0731	0.126*	0.50
H26D	0.3039	0.5038	−0.0079	0.126*	0.50
C27'	0.304 (6)	0.365 (4)	0.016 (3)	0.097 (8)	0.50
H27C	0.3790	0.3749	0.0396	0.116*	0.50
H27D	0.3072	0.3087	−0.0119	0.116*	0.50
C28'	0.204 (4)	0.363 (4)	0.067 (3)	0.099 (7)	0.50
H28C	0.1959	0.3007	0.0862	0.119*	0.50
H28D	0.2195	0.4061	0.1047	0.119*	0.50
C25'	0.134 (5)	0.457 (3)	−0.013 (2)	0.102 (8)	0.50
H25C	0.1159	0.5176	0.0071	0.122*	0.50
H25D	0.0909	0.4506	−0.0565	0.122*	0.50
O3'	0.1045 (16)	0.3878 (11)	0.0316 (9)	0.097 (5)	0.50

### Atomic displacement parameters (Å^2^)

**Table:**

	*U*^11^	*U*^22^	*U*^33^	*U*^12^	*U*^13^	*U*^23^
Ni1	0.0352 (3)	0.0362 (3)	0.0333 (3)	−0.0028 (2)	0.0057 (2)	−0.0022 (2)
Ni2	0.0407 (3)	0.0463 (4)	0.0423 (3)	−0.0016 (3)	0.0050 (2)	0.0095 (2)
C1	0.050 (3)	0.065 (3)	0.057 (3)	−0.013 (2)	0.010 (2)	−0.008 (2)
C2	0.043 (3)	0.085 (4)	0.071 (3)	−0.016 (3)	0.015 (3)	−0.002 (3)
C3	0.040 (3)	0.086 (4)	0.054 (3)	0.002 (3)	0.015 (2)	0.000 (3)
C4	0.045 (3)	0.058 (3)	0.037 (2)	0.009 (2)	0.0071 (19)	0.003 (2)
C5	0.053 (3)	0.064 (3)	0.056 (3)	0.018 (3)	0.013 (2)	−0.010 (2)
C6	0.070 (4)	0.059 (3)	0.053 (3)	0.014 (3)	0.007 (3)	−0.014 (2)
C7	0.056 (3)	0.040 (3)	0.040 (2)	0.001 (2)	−0.002 (2)	−0.0076 (19)
C8	0.072 (4)	0.042 (3)	0.053 (3)	−0.003 (3)	−0.003 (3)	−0.015 (2)
C9	0.055 (3)	0.050 (3)	0.068 (3)	−0.011 (2)	−0.009 (3)	−0.002 (2)
C10	0.041 (3)	0.046 (3)	0.048 (2)	−0.008 (2)	0.002 (2)	−0.006 (2)
C11	0.040 (2)	0.036 (2)	0.036 (2)	0.0040 (19)	0.0036 (18)	−0.0009 (18)
C12	0.036 (2)	0.046 (3)	0.033 (2)	0.0026 (19)	0.0032 (18)	−0.0014 (19)
C13	0.049 (3)	0.052 (3)	0.045 (2)	0.007 (2)	0.002 (2)	−0.001 (2)
C14	0.039 (3)	0.066 (3)	0.066 (3)	0.010 (2)	0.007 (2)	−0.005 (3)
C15	0.043 (3)	0.078 (4)	0.060 (3)	−0.001 (3)	0.025 (2)	−0.005 (3)
C16	0.046 (3)	0.052 (3)	0.049 (3)	−0.005 (2)	0.012 (2)	−0.004 (2)
C17	0.066 (3)	0.068 (3)	0.048 (3)	−0.017 (3)	0.017 (2)	0.004 (3)
C18	0.077 (4)	0.062 (3)	0.039 (2)	−0.007 (3)	0.007 (2)	0.010 (2)
C19	0.056 (3)	0.045 (3)	0.041 (2)	−0.006 (2)	−0.001 (2)	0.000 (2)
C20	0.076 (4)	0.056 (3)	0.054 (3)	0.005 (3)	−0.011 (3)	0.014 (2)
C21	0.055 (3)	0.074 (4)	0.072 (3)	0.009 (3)	−0.019 (3)	0.002 (3)
C22	0.036 (3)	0.064 (3)	0.056 (3)	0.006 (2)	−0.001 (2)	−0.001 (2)
C23	0.043 (2)	0.037 (2)	0.034 (2)	−0.0033 (19)	0.0004 (18)	−0.0006 (18)
C24	0.036 (2)	0.037 (2)	0.039 (2)	−0.0033 (18)	0.0063 (18)	−0.0044 (18)
C29	0.044 (3)	0.046 (3)	0.052 (3)	−0.004 (2)	−0.002 (2)	0.004 (2)
C30	0.051 (3)	0.049 (3)	0.035 (2)	−0.005 (2)	0.004 (2)	0.007 (2)
C31	0.042 (3)	0.060 (3)	0.045 (2)	−0.001 (2)	0.001 (2)	0.004 (2)
C32	0.051 (3)	0.065 (3)	0.052 (3)	−0.001 (3)	0.004 (2)	0.016 (2)
N1	0.038 (2)	0.0334 (19)	0.0398 (18)	−0.0016 (15)	0.0025 (15)	0.0004 (15)
N2	0.038 (2)	0.048 (2)	0.0403 (18)	−0.0111 (17)	0.0066 (15)	−0.0053 (16)
N3	0.0343 (19)	0.040 (2)	0.0347 (17)	0.0029 (16)	0.0027 (14)	0.0003 (15)
N4	0.037 (2)	0.038 (2)	0.0459 (19)	−0.0004 (16)	0.0036 (16)	−0.0017 (16)
N5	0.068 (3)	0.062 (3)	0.064 (2)	−0.005 (2)	0.010 (2)	0.024 (2)
N6	0.054 (3)	0.063 (3)	0.062 (2)	0.009 (2)	0.011 (2)	0.009 (2)
N7	0.075 (3)	0.079 (3)	0.064 (3)	−0.016 (3)	0.011 (2)	0.026 (2)
N8	0.059 (3)	0.104 (4)	0.112 (4)	0.027 (3)	0.018 (3)	0.042 (3)
O1	0.100 (3)	0.067 (2)	0.0558 (19)	−0.003 (2)	0.0047 (19)	0.0020 (17)
O2	0.079 (2)	0.062 (2)	0.070 (2)	−0.0121 (18)	0.0100 (18)	−0.0100 (18)
O4	0.095 (3)	0.067 (2)	0.064 (2)	−0.014 (2)	−0.0115 (19)	0.0090 (18)
C25	0.11 (2)	0.109 (10)	0.092 (7)	0.014 (10)	0.004 (10)	0.025 (8)
C26	0.099 (8)	0.131 (18)	0.085 (12)	0.029 (11)	0.017 (7)	0.027 (10)
C27	0.077 (5)	0.122 (13)	0.09 (2)	0.020 (8)	0.015 (14)	0.009 (12)
C28	0.078 (5)	0.135 (8)	0.084 (18)	0.023 (5)	0.017 (8)	0.027 (11)
O3	0.112 (15)	0.155 (19)	0.18 (2)	0.059 (13)	0.037 (12)	0.055 (13)
C26'	0.099 (8)	0.131 (18)	0.085 (12)	0.029 (11)	0.017 (7)	0.027 (10)
C27'	0.077 (5)	0.122 (13)	0.09 (2)	0.020 (8)	0.015 (14)	0.009 (12)
C28'	0.078 (5)	0.135 (8)	0.084 (18)	0.023 (5)	0.017 (8)	0.027 (11)
C25'	0.11 (2)	0.109 (10)	0.092 (7)	0.014 (10)	0.004 (10)	0.025 (8)
O3'	0.069 (7)	0.111 (12)	0.111 (10)	0.004 (8)	0.000 (6)	0.046 (8)

### Geometric parameters (Å, °)

**Table:**

Ni1—O2	2.104 (3)	C19—C20	1.405 (6)
Ni1—N2	2.108 (3)	C19—C23	1.410 (5)
Ni1—N3	2.109 (3)	C20—C21	1.364 (7)
Ni1—N1	2.110 (3)	C20—H20	0.9300
Ni1—N4	2.118 (3)	C21—C22	1.396 (6)
Ni1—O1	2.127 (3)	C21—H21	0.9300
Ni2—C31	1.862 (5)	C22—N4	1.318 (5)
Ni2—C30	1.873 (5)	C22—H22	0.9300
Ni2—C32	1.875 (5)	C23—N4	1.360 (5)
Ni2—C29	1.878 (5)	C23—C24	1.439 (5)
C1—N2	1.331 (5)	C24—N3	1.365 (5)
C1—C2	1.387 (6)	C29—N5	1.149 (5)
C1—H1	0.9300	C30—N6	1.147 (5)
C2—C3	1.359 (6)	C31—N7	1.154 (5)
C2—H2	0.9300	C32—N8	1.135 (6)
C3—C4	1.412 (6)	O1—H1A	0.9004
C3—H3	0.9300	O1—H1B	0.8553
C4—C12	1.402 (5)	O2—H2B	0.7882
C4—C5	1.424 (6)	O2—H2A	0.8229
C5—C6	1.341 (6)	O4—H4B	0.8335
C5—H5	0.9300	O4—H4A	0.8377
C6—C7	1.428 (6)	C25—O3	1.356 (18)
C6—H6	0.9300	C25—C26	1.512 (18)
C7—C11	1.390 (5)	C25—H25A	0.9700
C7—C8	1.398 (6)	C25—H25B	0.9700
C8—C9	1.361 (6)	C26—C27	1.496 (19)
C8—H8	0.9300	C26—H26A	0.9700
C9—C10	1.394 (6)	C26—H26B	0.9700
C9—H9	0.9300	C27—C28	1.517 (16)
C10—N1	1.325 (5)	C27—H27A	0.9700
C10—H10	0.9300	C27—H27B	0.9700
C11—N1	1.366 (5)	C28—O3	1.365 (19)
C11—C12	1.442 (5)	C28—H28A	0.9700
C12—N2	1.356 (5)	C28—H28B	0.9700
C13—N3	1.315 (5)	C26'—C27'	1.495 (19)
C13—C14	1.396 (6)	C26'—C25'	1.513 (18)
C13—H13	0.9300	C26'—H26C	0.9700
C14—C15	1.359 (6)	C26'—H26D	0.9700
C14—H14	0.9300	C27'—C28'	1.516 (16)
C15—C16	1.408 (6)	C27'—H27C	0.9700
C15—H15	0.9300	C27'—H27D	0.9700
C16—C24	1.397 (5)	C28'—O3'	1.365 (19)
C16—C17	1.439 (6)	C28'—H28C	0.9700
C17—C18	1.341 (7)	C28'—H28D	0.9700
C17—H17	0.9300	C25'—O3'	1.356 (18)
C18—C19	1.430 (6)	C25'—H25C	0.9700
C18—H18	0.9300	C25'—H25D	0.9700
			
O2—Ni1—N2	94.81 (13)	C22—C21—H21	120.3
O2—Ni1—N3	91.89 (12)	N4—C22—C21	123.1 (4)
N2—Ni1—N3	170.74 (13)	N4—C22—H22	118.4
O2—Ni1—N1	173.25 (13)	C21—C22—H22	118.4
N2—Ni1—N1	78.57 (12)	N4—C23—C19	122.6 (4)
N3—Ni1—N1	94.55 (12)	N4—C23—C24	117.9 (3)
O2—Ni1—N4	90.46 (12)	C19—C23—C24	119.4 (4)
N2—Ni1—N4	94.27 (13)	N3—C24—C16	123.4 (4)
N3—Ni1—N4	79.26 (12)	N3—C24—C23	117.0 (3)
N1—Ni1—N4	88.75 (12)	C16—C24—C23	119.6 (4)
O2—Ni1—O1	91.88 (13)	N5—C29—Ni2	178.5 (4)
N2—Ni1—O1	90.27 (13)	N6—C30—Ni2	175.9 (4)
N3—Ni1—O1	95.91 (13)	N7—C31—Ni2	178.7 (4)
N1—Ni1—O1	89.48 (13)	N8—C32—Ni2	179.2 (5)
N4—Ni1—O1	174.71 (13)	C10—N1—C11	117.5 (3)
C31—Ni2—C30	87.64 (18)	C10—N1—Ni1	128.5 (3)
C31—Ni2—C32	89.77 (19)	C11—N1—Ni1	114.0 (2)
C30—Ni2—C32	177.23 (18)	C1—N2—C12	117.4 (4)
C31—Ni2—C29	178.2 (2)	C1—N2—Ni1	128.9 (3)
C30—Ni2—C29	92.06 (18)	C12—N2—Ni1	113.7 (3)
C32—Ni2—C29	90.56 (19)	C13—N3—C24	117.0 (3)
N2—C1—C2	123.1 (4)	C13—N3—Ni1	129.9 (3)
N2—C1—H1	118.5	C24—N3—Ni1	113.2 (2)
C2—C1—H1	118.5	C22—N4—C23	118.1 (4)
C3—C2—C1	119.7 (5)	C22—N4—Ni1	129.3 (3)
C3—C2—H2	120.1	C23—N4—Ni1	112.5 (3)
C1—C2—H2	120.1	Ni1—O1—H1A	107.6
C2—C3—C4	119.5 (4)	Ni1—O1—H1B	119.2
C2—C3—H3	120.2	H1A—O1—H1B	101.6
C4—C3—H3	120.2	Ni1—O2—H2B	142.3
C12—C4—C3	116.7 (4)	Ni1—O2—H2A	105.8
C12—C4—C5	119.5 (4)	H2B—O2—H2A	101.7
C3—C4—C5	123.8 (4)	H4B—O4—H4A	97.2
C6—C5—C4	121.0 (4)	O3—C25—C26	113 (4)
C6—C5—H5	119.5	O3—C25—H25A	108.9
C4—C5—H5	119.5	C26—C25—H25A	108.9
C5—C6—C7	121.4 (4)	O3—C25—H25B	108.9
C5—C6—H6	119.3	C26—C25—H25B	108.9
C7—C6—H6	119.3	H25A—C25—H25B	107.7
C11—C7—C8	116.8 (4)	C27—C26—C25	97 (5)
C11—C7—C6	118.9 (4)	C27—C26—H26A	112.3
C8—C7—C6	124.2 (4)	C25—C26—H26A	112.3
C9—C8—C7	119.9 (4)	C27—C26—H26B	112.3
C9—C8—H8	120.1	C25—C26—H26B	112.3
C7—C8—H8	120.1	C26—C27—C28	111 (5)
C8—C9—C10	119.7 (4)	C26—C27—H27A	109.4
C8—C9—H9	120.2	C28—C27—H27A	109.4
C10—C9—H9	120.2	C26—C27—H27B	109.4
N1—C10—C9	122.4 (4)	C28—C27—H27B	109.4
N1—C10—H10	118.8	H27A—C27—H27B	108.0
C9—C10—H10	118.8	O3—C28—C27	104 (5)
N1—C11—C7	123.6 (4)	O3—C28—H28A	111.1
N1—C11—C12	116.1 (3)	C27—C28—H28A	111.1
C7—C11—C12	120.2 (4)	O3—C28—H28B	111.1
N2—C12—C4	123.5 (4)	C27—C28—H28B	111.1
N2—C12—C11	117.5 (3)	H28A—C28—H28B	109.0
C4—C12—C11	119.0 (4)	C25—O3—C28	113 (4)
N3—C13—C14	123.8 (4)	C27'—C26'—C25'	107 (5)
N3—C13—H13	118.1	C27'—C26'—H26C	110.4
C14—C13—H13	118.1	C25'—C26'—H26C	110.4
C15—C14—C13	119.2 (4)	C27'—C26'—H26D	110.4
C15—C14—H14	120.4	C25'—C26'—H26D	110.4
C13—C14—H14	120.4	H26C—C26'—H26D	108.6
C14—C15—C16	119.4 (4)	C26'—C27'—C28'	97 (5)
C14—C15—H15	120.3	C26'—C27'—H27C	112.4
C16—C15—H15	120.3	C28'—C27'—H27C	112.4
C24—C16—C15	117.1 (4)	C26'—C27'—H27D	112.4
C24—C16—C17	119.7 (4)	C28'—C27'—H27D	112.4
C15—C16—C17	123.2 (4)	H27C—C27'—H27D	110.0
C18—C17—C16	120.5 (4)	O3'—C28'—C27'	108 (5)
C18—C17—H17	119.8	O3'—C28'—H28C	110.0
C16—C17—H17	119.8	C27'—C28'—H28C	110.0
C17—C18—C19	121.7 (4)	O3'—C28'—H28D	110.0
C17—C18—H18	119.1	C27'—C28'—H28D	110.0
C19—C18—H18	119.1	H28C—C28'—H28D	108.4
C20—C19—C23	117.2 (4)	O3'—C25'—C26'	107 (4)
C20—C19—C18	123.7 (4)	O3'—C25'—H25C	110.4
C23—C19—C18	119.1 (4)	C26'—C25'—H25C	110.4
C21—C20—C19	119.4 (4)	O3'—C25'—H25D	110.4
C21—C20—H20	120.3	C26'—C25'—H25D	110.4
C19—C20—H20	120.3	H25C—C25'—H25D	108.6
C20—C21—C22	119.4 (5)	C25'—O3'—C28'	107 (4)
C20—C21—H21	120.3		

### Hydrogen-bond geometry (Å, °)

**Table:**

*D*—H···*A*	*D*—H	H···*A*	*D*···*A*	*D*—H···*A*
O4—H4A···N8^i^	0.84	2.02	2.857 (6)	173
O2—H2A···O4^ii^	0.82	2.30	3.116 (5)	173
O2—H2B···N5^iii^	0.79	2.60	3.154 (5)	128
O1—H1B···N5^iii^	0.86	2.63	3.377 (6)	147
O1—H1B···N5^iii^	0.86	2.63	3.377 (6)	147
O1—H1A···N6^iv^	0.90	2.39	3.250 (5)	161
O4—H4B···N7	0.83	1.99	2.804 (5)	167

Symmetry codes: (i) −*x*+3/2, *y*+1/2, −*z*+3/2; (ii) −*x*+3/2, *y*−1/2, −*z*+1/2; (iii) −*x*+1, −*y*, −*z*+1; (iv) *x*+1/2, −*y*+1/2, *z*−1/2.
